# Model minority stereotype, school and socioeconomic achievement, and mental health of Filipino American and Korean American youth

**DOI:** 10.1111/jora.70146

**Published:** 2026-01-20

**Authors:** Michael Park, Yuanyuan Yang, Bryan Gu, Yoonsun Choi, Hyung Chol Yoo

**Affiliations:** ^1^ Rutgers University School of Social Work New Brunswick New Jersey USA; ^2^ University of Oklahoma Anne and Henry Zarrow School of Social Work Norman Oklahoma USA; ^3^ The University of Chicago Crown Family School of Social Work, Policy and Practice Chicago Illinois USA; ^4^ Arizona State University School of Social Transformation Tempe Arizona USA

**Keywords:** Asian American youth, mental health, model minority stereotype

## Abstract

Asian Americans are often stereotyped as model minorities—hardworking (achievement aspect) and unaffected by socioeconomic barriers (mobility aspect). However, the impact of these stereotypes on mental health remains unclear. This study is the first to examine longitudinally how internalizing these stereotypes relates to mental health, as moderated by grade point average (GPA), parental education, and household income among Asian American subgroups. Using a three‐wave panel study of 610 Filipino and Korean American youth (*M*_*age*_ *.*_*Wave2*_ = 16 years; 52% female), findings reveal that each aspect of the stereotype has distinct impacts on mental health, with variations by ethnicity, academic performance, and family socioeconomic status. These results contribute to identifying profiles of youth at risk for mental health challenges and guiding targeted mental health interventions for minoritized youth.

The Asian American population is one of the fastest growing in the United States and is projected to be the largest immigrant group in the U.S. by 2055 (Budiman & Ruiz, 2021). Despite their increasing presence, there remains a relative dearth of research addressing the mental health concerns of Asian Americans (Đoàn et al., 2019). This lack of attention is particularly alarming given their high rates of mental health struggles (Kim et al., 2015) and notably low utilization of mental health services (Substance Abuse and Mental Health Services Administration, 2019).

These inequities gesture toward the study of distinct factors associated with Asian American communities, such as racial stereotypes, as significant determinants of Asian Americans' mental health experiences. For example, Asian Americans are often stereotyped as a model minority, with reputations for high academic achievement and socioeconomic attainment, distinguished from other racially minoritized groups (Yoo et al., 2010). On its own, the model minority stereotype has displayed negative effects on the mental health of Asian American youth: Internalizing the stereotype can exert undue pressure on youth to conform to the stereotype's expectations (Lee & Hong, 2020). Internalization of the stereotype may even deter youth from seeking mental health services, as such help‐seeking may be viewed as a concession incongruent with the stereotype (Kim & Lee, 2014).

However, research also finds evidence that associates the model minority stereotype with positive effects, for example, promoting academic adjustment (Thompson & Kiang, 2010) and protecting against experiences of discrimination (Kiang et al., 2016). Recent studies suggest that these mixed findings may result from interactions between the model minority stereotype and other contextual factors: The consequences of internalizing the stereotype may depend on the level of exposure to racism (Tummala‐Narra et al., 2018), the concurrent salience of other forms of stereotype (Park et al., 2021), the racial composition of school settings (Atkin et al., 2018), or an individual's place of birth (Park et al., 2021). These recent studies underscore the complexity of the stereotype's impact and the importance of considering multiple contexts in its study.

To build on this understanding of contextual factors, we examine two elements central to the model minority stereotype: academic achievement and family socioeconomic status (SES). As model minorities, Asian Americans are stereotyped as smart, hardworking, and achievement‐oriented (achievement aspect), and as a group that faces fewer barriers to upward socioeconomic mobility than other racially minoritized groups, if any (mobility aspect; Yoo et al., 2010). Academic achievement and family SES are then intrinsically linked to these two aspects of the model minority stereotype: academic achievement is often cited as evidence of the achievement dimension, reinforcing the perception of Asian Americans as intelligent and industrious; greater SES, in turn, affirms the unrestricted mobility dimension.

Despite these connections, no previous study has explored how academic achievement and SES together moderate the relationship between the internalization of the model minority stereotype and mental health outcomes. Some earlier studies have individually investigated the moderating roles of either academic achievement or SES, although findings have been mixed. Le et al. (2024) found subjective SES to have no significant moderating effect in the relationships between both aspects of the model minority stereotype and psychological distress, whereas Yoo et al. (2015) identified GPA as a protective factor in the relationship between both aspects of the stereotype and psychological adjustment. Notably, Le et al. (2024) used subjective family SES; no studies to date have used objective measures of family SES as a moderator of the model minority stereotype's effects on youth outcomes. Drawing on cognitive dissonance theory (Festinger, 1957), we expect that Asian American students whose academic achievement or family SES contradict the model minority stereotype may face greater distress (Cocchiara & Quick, 2004). Conversely, those whose academic performance and socioeconomic background align with the model minority stereotype may experience minimal psychological conflict and may even experience benefits from internalization. However, empirical research confirming these interactions—and further exploring the complexities of satisfying some but not all dimensions of the stereotype—remains limited.

The current longitudinal study addresses this research gap by examining how GPA and two indicators of family SES (parental educational attainment and household income) moderate the impact of the internalized model minority stereotype on mental health outcomes among Asian American youth. By analyzing both between‐subjects effects (across individuals) and within‐subjects effects (over time), this investigation offers unique insights into potential longitudinal impacts of the stereotype. Moreover, by comparing different ethnic subgroups (Filipino Americans vs. Korean Americans) and examining contextual factors reflecting both dimensions of the model minority stereotype (GPA and objective SES), our research aims to deepen understanding of how the model minority stereotype affects mental health among Asian American youth. Ultimately, these findings will help identify profiles of youth at risk for mental health challenges and inform the development of more targeted intervention strategies for racially minoritized youth experiencing mental distress.

## CONCEPTUAL FRAMEWORK

A substantial body of research documents the widespread prevalence of mental health challenges during adolescence, especially those related to depression and suicidality, which often intensify as adolescents progress through this developmental period (Hankin et al., 1998; Substance Abuse and Mental Health Services Administration, 2022). Asian American adolescents are disproportionately affected, reporting higher rates of major depressive episodes than their peers from other racialized groups (Substance Abuse and Mental Health Services Administration, 2017). Moreover, suicide was found to be the leading cause of death among Asian Americans aged 15 to 24—a unique pattern not observed for most other racial or ethnic populations (Kochanek et al., 2019).

For Asian American adolescents, mental health risks are further amplified by a combination of structural and sociodemographic factors, including racial minoritization and associated experiences of racial stereotyping (Park et al., 2021). Although there is a dearth of conceptual or empirical research on how Asian American adolescents internalize the model minority stereotype across developmental stages, emerging studies have suggested that Asian American adolescents may internalize this stereotype in varying degrees (Park, 2020). This variability is a key consideration in understanding the diverse mental health outcomes within this population.

To better understand how internalized racial stereotypes shape mental health outcomes, one can turn to cognitive dissonance theory (Festinger, 1957), a psychological framework that provides insight into how individuals manage conflicting beliefs or experiences. Cognitive dissonance theory suggests that individuals experience psychological discomfort when their beliefs, attitudes, or expectations conflict with their actual behaviors or experiences. The model minority stereotype frames academic success and socioeconomic attainment as normative; when students struggle academically or face socioeconomic challenges, the dissonance between the internalized narrative of the stereotype and their lived realities may lead to psychological distress (Cocchiara & Quick, 2004). In contrast, students whose experiences more closely align with the stereotype's narrative—such as those who perform well academically or enjoy greater SES with fewer structural barriers—may find that it affirms their self‐concept and subsequently supports their well‐being. Cognitive dissonance may then help to explain why the mental health effects of the model minority stereotype could vary depending on students’ academic and socioeconomic contexts.

Both conceptual studies (Coll et al., 1996) and empirical research (Park et al., 2021) suggest that the impact of racial stereotypes on minority youth can also vary by ethnicity. Consequently, this study focuses on Filipino Americans and Korean Americans as two Asian American ethnic subgroups that may experience the model minority stereotype differently. Korean Americans, as part of the broader East Asian American subgroup, are frequently linked to the “model minority” stereotype (Kim, 1999), which contributes to elevated expectations placed upon them by both society and themselves (Ochoa, 2013). As a result, endorsing the achievement aspect of the stereotype—which highlights self‐discipline and individual effort—may amplify the academic and social pressures they experience (Yoo et al., 2015). In contrast, internalizing the belief that Asian Americans are relatively exempt from racial discrimination compared with other racially minoritized groups (the mobility aspect) may reduce some of the strain associated with meeting such high standards (Yoo et al., 2015). Filipino Americans, on the other hand, often experience a bivalent racial identity that complicates their visibility as Asian Americans to others and themselves. For example, their phenotype and cultural practices—such as religious traditions, family structures, and cuisine—often align more closely with those of Latino Americans, leading to frequent misidentification (Ocampo, 2016). Additionally, the Philippines’ long colonial history under Spain further reinforces these perceived cultural ties with Latin American countries. As a result, Filipino Americans are less likely to be stereotyped as model minorities (Nadal, 2008). For this group, then, internalizing the ostensibly positive traits of the model minority stereotype may offer psychological benefits (Kiang et al., 2016). However, for both ethnic groups, the psychological consequences of the model minority stereotype may be contingent upon individuals' academic performance and socioeconomic positioning. By comparing responses to the model minority stereotype between these two groups, this study acknowledges the heterogeneity of the Asian American community and the experiences of its constituents. Korean and Filipino Americans represent two communities with distinct characteristics that shape both the consideration shown toward each of these groups as model minorities and the individual stereotype experiences of community members. In making such comparisons, this research aims to provide a more nuanced understanding of how racial stereotypes and contextual factors relevant to the stereotype (e.g., academic achievement and family SES) shape the mental health of Asian American subgroups.

## MODEL MINORITY STEREOTYPE AND MENTAL HEALTH

The model minority stereotype can be understood to consist of two aspects: The first is the achievement orientation stereotype (the achievement aspect), which holds that Asian Americans succeed because of their strong work ethic and commitment to achievement. The second is the unrestricted mobility stereotype (the mobility aspect), which suggests that Asian Americans achieve greater SES because they do not encounter the same barriers to upward mobility that other racial minority groups face (Yoo et al., 2010).

These two aspects have been found to have different effects on Asian American young adults. For example, Yoo et al. (2010), in a study of Asian American college students, found that both aspects of the stereotype were associated with negative outcomes, but in different domains: The achievement aspect was significantly related to performance difficulty, while the mobility aspect was significantly linked to general and somatic distress. In contrast, a later study by Yoo et al. (2015) with Asian American adolescents showed that the achievement aspect was associated with greater stress related to academic expectations, while the mobility aspect was associated with lower levels of stress. Although both studies included Filipino American and Korean American participants alongside other Asian American subgroups, the impact of ethnic subgroup membership was not examined or controlled for in the analyses.

Subsequent research has revealed more nuanced and subgroup‐specific patterns. Using cross‐sectional data from 308 Filipino American and 340 Korean American youth, Park et al. (2021) found even more complex impacts of the model minority stereotype: Although neither aspect of the model minority stereotype showed direct effects on internalizing outcomes, significant interaction effects emerged between the two aspects. Specifically, among Korean American youth, those who highly internalized both aspects of the stereotype reported higher levels of negative affect and depressive symptoms. Furthermore, the achievement aspect amplified the negative effects of the perpetual foreigner stereotype on negative affect and depressive symptoms for Korean American emerging adults (age 18 and above), but not among adolescents (under 18). In contrast, the mobility aspect showed protective qualities: Its internalization mitigated the harmful effects of the perpetual foreigner stereotype on negative affect and depressive symptoms among Filipino American emerging adults and among Korean American participants, regardless of their age groups. Qualitative research with Asian American college students further supports these differential effects. While many students reported distress from trying to meet model minority achievement expectations, others found support in the mobility aspect's race‐evasive affirmation of meritocracy (Assalone & Fann, 2017; Lee et al., 2009).

## ACADEMIC PERFORMANCE, SES, AND MENTAL HEALTH

Given the centrality of both academic achievement and SES within the model minority stereotype, understanding how these individual differences may impact young adults' experience of the model minority stereotype is critical. Research has shown significant associations between academic achievement and mental health in the general population (Bas, 2021) as well as among Asian American youth (Whaley & Noel, 2013). For Asian American young adults, academic matters can be one of the primary concerns underlying mental distress. Wong and Maffini (2011), in a qualitative study of 293 Asian Americans at a large public West Coast university, identified academic problems as one of three major events that preceded suicidal thoughts. Whaley and Noel (2013) found that Asian American adolescents with lower GPAs reported higher rates of depressed mood and suicidal thoughts. Studies have also shown that lower GPA was associated with more externalizing problem behaviors, such as substance use and violent behavior (Choi, 2007; Whaley & Noel, 2013).

With regard to SES, despite extant research suggesting a clear link between SES—including parental educational attainment (Chen et al., 2022) and household income (Cooper & Stewart, 2021)—and mental health outcomes in the general youth population, research specifically examining mental health outcomes of Asian American youth is limited (see Qin et al., 2017, for reviews). SES is often used as a control variable rather than a central focus of the investigation, and among this research, many studies have found no significant effects of SES on Asian American mental health outcomes (Qin et al., 2017). Some authors have suggested that the nonsignificant direct findings may stem from differences in the significance of SES indicators for Asian Americans compared with other racial groups. For example, Gong et al. (2012) suggested that for Asian Americans, subjective rather than objective SES indicators may be more critical determinants of mental health outcomes. The lack of significant associations with objective SES indicators may also be attributed to methodological issues, such as the aggregation of data across diverse Asian American ethnic subgroups, which can obscure meaningful within‐group differences. For example, Kim et al. (2012) found poverty to be significantly related to self‐reported mental health, but only in Vietnamese populations, who reported the highest rate of poverty among the sampled ethnic groups. This heterogeneity underscores the importance of examining objective SES indicators in relation to mental health across distinct Asian American ethnic groups. Accordingly, the present study includes two objective indicators—parental educational attainment and household income—as proxies for SES.

## ACADEMIC PERFORMANCE AND SES AS MODERATORS

Limited studies have examined academic performance or SES as moderators in the relation between the internalized model minority stereotype and mental health. For example, Yoo et al. (2015), in a study of 155 Asian American high school adolescents, examined the moderating role of GPA in the association between the two aspects of the internalized model minority stereotype and a set of psychological adjustment outcomes, including affective distress, somatic distress, performance difficulty, and expectations stress. They found protective moderating effects of GPA on the association between the achievement aspect and performance difficulty, as well as between the mobility aspect and affective distress. The authors posited that these findings reflect a dual modality within the model minority stereotype, such that those who satisfy the stereotype experience greater benefit, while those who do not meet the expectations struggle from this cognitive dissonance. The present study builds on this work by testing these conclusions across time, ethnicities, and family SES.

Regarding family SES, for example, in a study of 365 low‐income Asian American adults (*M*_*age*_ = 37 years, SD = 16.19), the mobility aspect was related to less psychological distress, while the achievement aspect was not a significant predictor (Le et al., 2024). However, this study did not find a significant moderating effect of subjective SES in the relationships between both aspects of the stereotype and psychological distress. These nonsignificant findings may stem from the study's limited consideration of academic performance, from its aggregation of different ethnic groups, or from its use of subjective rather than objective SES. The present study expands on this research by examining these relationships using parental educational attainment and family household income across different ethnic communities, while also considering the role of academic performance.

## PRESENT STUDY

Using three waves of longitudinal data (2016–2022) from Filipino American and Korean American youth in the midwestern United States, this study first investigates the direct effects of the internalized model minority stereotype (achievement and mobility aspects), GPA, and SES (parental educational attainment and household income) on mental health outcomes, specifically depressive symptoms and suicidal thoughts. Then, it investigates how the longitudinal relations between each aspect of the model minority stereotype and these mental health outcomes are moderated by GPA and SES across Filipino American and Korean American youth. Finally, it explores whether and how these moderating effects of GPA further vary by SES, and vice versa.

First, regarding the direct effects model, prior conceptual and empirical research led us to expect that both higher GPA and higher SES would be associated with fewer mental health problems for both ethnic groups. Regarding the model minority stereotype, we predicted that the effects of the stereotype would differ across Asian American subgroups due to their distinct sociocultural backgrounds and racial stereotyping experiences. Specifically, for Filipino American youth, we hypothesized that both the achievement and mobility aspects of the model minority stereotype would serve protective functions. Internalizing these stereotypes could provide a sense of positive group identity for a community that has historically been less associated with the model minority stereotype and its favorable traits, such as intelligence and a strong work ethic. In such cases, the psychological benefits of this identity may outweigh the potential negative consequences of the stereotype. For Korean American youth, we predicted a more complex pattern: Internalizing the achievement aspect of the stereotype would increase mental health problems due to added pressure to meet heightened academic and social expectations typically placed on East Asian Americans, while internalizing the mobility aspect would help reduce psychological distress by fostering a sense of potential socioeconomic advancement despite racial barriers.

Second, with respect to the two‐way interaction model, we hypothesized that GPA or SES would moderate these relationships, such that lower GPA or lower SES would amplify the negative effects and weaken the positive effects of the stereotype, due to the cognitive dissonance resulting from the discrepancy between the high expectations associated with the stereotype and individuals' actual academic and socioeconomic achievement. Specifically, for Korean American youth, we predicted that lower GPA or lower SES would exacerbate the psychological burden of the achievement aspect and reduce any stress‐buffering effects of the mobility aspect. While both aspects of the model minority stereotype were predicted to generally serve protective functions for Filipino American youth, we predicted that these beneficial effects would be attenuated among those with lower GPA or lower SES.

Finally, regarding the three‐way interaction model, although no prior research exists to provide guidance, we expected that for Korean American youth, the psychological burden of the achievement aspect would be particularly pronounced, and any protective effects of the mobility aspect would be diminished among those with both lower GPA and lower SES. Similarly, for Filipino American youth, the generally protective impact of internalized stereotypes was anticipated to be weakest among individuals with lower standing on both academic and socioeconomic dimensions.

## METHODS

### Participants

The data used in this study were sourced from the Midwest Longitudinal Study of Asian American Families. This longitudinal survey involved Filipino American and Korean American children and their parents residing in the Chicago metropolitan area. While the complete study comprises four waves of panel data, this analysis used child data from Waves 2 to 4, as the internalized model minority stereotype measure was first introduced in Wave 2. The initial wave, conducted in 2014, included 378 Filipino American youth and 376 of their parents, alongside 408 Korean American youth and 412 parents, culminating in a total sample size of 1574. Given that this study focuses on Asian American youth, this study exclusively analyzed data pertaining to the youth. The retention rates for Filipino American youth were 75% in 2016 (Wave 2; *n* = 282), 81% in 2018 (Wave 3; *n* = 308), and 70% in 2021 (Wave 4; *n* = 265). For Korean American youth, the rates were 80% in 2016 (Wave 2; *n* = 328), 83% in 2018 (Wave 3; *n* = 340), and 85% in 2021 (Wave 4; *n* = 347). The biological sex distribution in Wave 2 included 59.14% female among Filipino American youth and 47.38% female among Korean American youth. Approximately 72.24% of Filipino American youth and 60.55% of Korean American youth were United States‐born. At Wave 2, the average age was 16.71 years (SD = 1.87) for Filipino American youth and 16.39 years (SD = 1.85) for Korean American youth. On average, youth in both groups reported good overall physical health, and their parents typically held at least a college degree, as detailed in Table 1.

**TABLE 1:** Descriptive statistics.

Variables	FA	KA	Diff.	Trajectory		
				FA	KA	All
Demographic characteristics						
Sample sizes (W2) [*n* (%)]	282 (100%)	328 (100%)				
Sample sizes (W3) [*n* (%)]	308 (100%)	340 (100%)		N/A	N/A	N/A
Sample sizes (W4) [*n* (%)]	265 (100%)	347 (100%)				
Age (W2)	16.71 (1.87)	16.39 (1.85)	***			
Age (W3)	18.22 (1.84)	17.91 (1.89)	*	N/A	N/A	N/A
Age (W4)	21.55 (1.83)	21.24 (1.85)	*			
Female (W2) [*n* (%)]	165 (59.14%)	154 (47.38%)	*			
Female (W3) [*n* (%)]	177 (57.65%)	168 (46.56%)	**	N/A	N/A	N/A
Female (W4) [*n* (%)]	158 (59.62%)	173 (49.86%)	*			
General health (W2)	3.92 (0.84)	3.83 (0.88)	n.s.			
General health (W3)	3.75 (0.82)	3.70 (0.86)	n.s.	−***	−***	−***
General health (W4)	3.58 (0.88)	3.45 (0.91)	†			
Predictors						
Achievement aspect (W2)	3.41 (0.73)	3.33 (0.72)	†			
Achievement aspect (W3)	3.39 (0.77)	3.45 (0.72)	n.s.	−**	−*	−***
Achievement aspect (W4)	3.26 (0.80)	3.24 (0.76)	n.s.			
Mobility aspect (W2)	2.82 (0.67)	2.68 (0.66)	*			
Mobility aspect (W3)	2.86 (0.73)	2.67 (0.76)	**	−*	−***	−***
Mobility aspect (W4)	2.69 (0.78)	2.42 (0.71)	***			
GPA (W2)	3.53 (0.48)	3.52 (0.47)	n.s.			
GPA (W3)	3.36 (0.55)	3.44 (0.47)	†	−**	n.s.	−**
GPA (W4)	3.44 (0.46)	3.51 (0.44)	†			
College degree (W2) [*n* (%)]	267 (80.52%)	319 (62.70%)	***			
College degree (W3) [*n* (%)]	233 (80.34%)	201 (61.74%)	***	N/A	N/A	N/A
College degree (W4) [*n* (%)]	194 (78.54%)	201 (60.00%)	***			
Higher income (W2) [*n* (%)]	180 (66.91%)	124 (39.74%)	***			
Higher income (W3) [*n* (%)]	194 (66.44%)	126 (39.25%)	***	N/A	N/A	N/A
Higher income (W4) [*n* (%)]	160 (64.00%)	126 (38.53%)	***			
Outcomes						
Depressive symptoms (W2)	1.89 (0.81)	1.97 (0.84)	n.s.			
Depressive symptoms (W3)	2.10 (0.82)	2.17 (0.84)	n.s.	+***	+***	+***
Depressive symptoms (W4)	2.27 (0.93)	2.29 (0.97)	n.s.			
Suicidal thoughts (W2) [*n* (%)]	35 (12.50%)	41 (12.69%)	n.s.			
Suicidal thoughts (W3) [*n* (%)]	49 (16.17%)	55 (16.37%)	n.s.	+	n.s.	+
Suicidal thoughts (W4) [*n* (%)]	46 (17.49%)	53 (15.32%)	n.s.			

Abbreviations: FA, Filipino American youth; KA, Korean American youth; minus sign (−), decreasing; plus sign (+), increasing; W, wave. ***p < .001; **p < .01; *p < .05; ^†^ *p* < .1.

### Recruitment and procedures

Participants were recruited during the initial data collection phase from various sources across the four main counties of the Chicago area. These sources included phone books, public and private schools, ethnic grocery stores, ethnic churches and temples, and ethnic community organizations. To qualify for the study, families had to meet two criteria at the baseline: (a) the adolescent should be between 12 and 17 years of age or a middle or high school student, and (b) the adolescent's biological mother must be of Korean or Filipino descent. The latter was required because the original study aimed to investigate the cultural process of parenting and parent–child relations, and mothers remain the primary caretaker in this population. In each household, one adolescent and their primary caregiver were invited to participate. As expected, primary caregivers were predominantly mothers (752 [95%]), with small proportions being fathers (26 [3%]) and other relatives or guardians (10 [1%]; e.g., grandparents, aunts, or legal guardians). Although the samples included a small proportion of multiracial families (higher among Filipino families), all participating primary caretakers were of Korean or Filipino descent. Recruitment materials were made available in Tagalog, Korean, and English. To express appreciation, youth participants received gift vouchers valued at $30, $70, and $50 during Waves 2, 3, and 4, respectively. More detailed information on recruitment for this longitudinal study has been published elsewhere (Choi et al., 2018).

### Measures

Unless specified differently, all measures used a 5‐point Likert scale for response options. A higher score represents a greater degree of the construct measured.

#### Depressive symptoms

Depressive symptoms for the two weeks prior to the survey were measured using 14 items drawn from the Children's Depression Inventory (Angold et al., 1995) and the Seattle Personality Questionnaire for Children (Kusche et al., 1988). Response options were on a 5‐point Likert scale ranging from 1 (*almost never*) to 5 (*almost always*). Questions such as “I didn't enjoy anything at all” and “I felt I was a bad person” were included. These measures showed high reliability, with α ranging from 0.94 to 0.95 across three waves for both Filipino American and Korean American youth.

#### Suicidal thoughts

Suicidal thoughts were assessed using a single binary question: “During the past 12 months, did you ever seriously think about committing suicide?” Responses were evaluated on a dichotomous scale (1 = *yes* and 0 = *no*).

#### Model minority stereotype

The model minority stereotype was assessed using two subscales developed from the internalization of the model minority myth measure (Yoo et al., 2010): the achievement and mobility aspects. Both aspects employed a 5‐point Likert‐type response format, ranging from 1 (*strongly disagree*) to 5 (*strongly agree*). The achievement aspect comprised 10 items (α ranging from 0.92 to 0.93 across three waves) and included statements such as “In comparison to other racial minorities (e.g., African‐Americans, Hispanics, Native Americans), Asian Americans generally perform better on standardized exams (e.g., SATs) because of their values regarding academic achievement” and “In comparison to other racial minorities (e.g., African‐Americans, Hispanics, Native Americans), Asian Americans make more money because they work harder.” The mobility aspect consisted of five items (α ranging from 0.77 to 0.83 across three waves) and included items such as “In comparison to other racial minorities (e.g., African‐Americans, Hispanics, Native Americans), Asian Americans are less likely to face barriers at work” and “In comparison to other racial minorities (e.g., African‐Americans, Hispanics, Native Americans), Asian Americans are less likely to experience racism in the United States.”

#### GPA

For participants aged 18 and older or who were enrolled in college at the time of the survey, we asked them to self‐report their overall GPA on a 4.0 scale for the most recent grading period. For those aged 17 years and younger, we asked them to self‐report their most recent letter grades in English, math, social studies, and science. These letter grades were then recoded into numeric values on a 4.0 scale (A = 4, B = 3, C = 2, D = 1, F = 0) and averaged across subjects to create an overall GPA score ranging from 0 to 4.

#### SES

We used parental educational attainment and household income as proxies for family SES. First, parents reported their highest level of education using the following categories: did not graduate high school, high school graduate or general educational development (GED), some college/associate degree, college graduate, and graduate degrees. This variable was then dichotomized, with 1 indicating a college degree or above (college degree and graduate degrees) and 0 indicating below a college degree (did not graduate high school, high school graduate, or GED, and some college/associate degree combined). Second, parents reported their total household income before taxes for the previous year (2014) using the following categories: (1) less than $25,000, (2) $25,000–$49,999, (3) $50,000–$74,999, (4) $75,000–$99,999, (5) $100,000–$149,999, and (6) $150,000 or more. This variable was subsequently dichotomized, with 1 indicating an income of $75,000 or higher and 0 indicating an income of $74,999 or lower. This household income cutoff is a widely accepted threshold among prior U.S. research examining the relationship between economic factors and mental health outcomes, distinguishing families with limited resources from those with greater economic resources (Hall et al., 2022; Kahneman & Deaton, 2010; Rudenstine et al., 2021).

### Control variables

The analysis controlled for demographic factors including baseline age, biological sex (male vs. female), nativity (United States‐born vs. foreign‐born), and self‐reported general health. To account for the varying effects of age over time, an interaction term between baseline age and wave was included, addressing the differential effects of age by cohort on the dependent variables.

### Analysis plan

This study adopted mixed‐effects regression models using Stata (Version 17), specifically employing random‐intercept modeling for each of the two dependent variables. Random‐intercept models enable the baseline outcome levels to vary among individuals. Additionally, these models accommodate predictors that either remain constant over time (time‐invariant, e.g., nativity) or change over time (time‐varying, e.g., the model minority stereotype).

### Between‐ and within‐subjects effects

To provide a more comprehensive evaluation of the study hypotheses, this study explored whether the time‐varying variables can be differentiated into between‐ and within‐subjects effects (Hedeker & Gibbons, 2006). Specifically, cross‐sectional (time‐invariant) data allow for the analysis of how predictors relate to outcomes across different individuals (between‐subjects effects). Conversely, longitudinal (time‐variant) data enable the analysis of how changes in predictors within an individual over time correlate with their outcomes over time (within‐subjects effects). Given the longitudinal design of this study, with three waves of cross‐sectional data, it was necessary to test whether the associations of interest were driven by between‐individual differences, within‐individual changes over time, or both. Without decomposing time‐varying predictors, one implicitly assumes that between‐ and within‐subjects effects are equivalent—an assumption that is often violated (Hedeker & Gibbons, 2006). Therefore, we first formally tested whether decomposition was warranted.

For example, to examine the effect of the achievement aspect of the stereotype on depressive symptoms, we employed the following procedure: First, we constructed a between‐subjects effect variable by averaging each subject's score on the achievement aspect subscale across the three waves. For the within‐subjects effect, we calculated the deviation of each subject's achievement aspect score in each wave from their average achievement aspect score over the three waves. We then conducted a likelihood ratio (LR) test to compare the fit of the models with and without this decomposition of the achievement aspect scores. If the LR test showed no significant difference, the simpler model without decomposition was used. In such cases, a significant effect of the achievement aspect in the nondecomposed model would suggest that the association holds both between individuals and within individuals over time. Conversely, if the LR test indicated a significant difference, the decomposed model was used to separately estimate between‐ and within‐subjects effects. For instance, if only the between‐subjects variable is significant, this would suggest that differences between individuals in the achievement aspect predict depressive symptoms. If only the within‐subjects variable is significant, it would suggest that changes in the achievement aspect within individuals over time are associated with changes in depressive symptoms. If both effects are significant, this would indicate that both types of relationships—between and within—contribute to the observed association, consistent with the results from a significant nondecomposed model.

### Stepwise analyses of the mixed‐effects regression models

Predictors were hierarchically included to test the study hypotheses through direct effects and interaction effects. Model 1 (the direct effect model) incorporates each aspect of the model minority stereotype variable, GPA, SES (parental educational attainment or household income), and control variables. Model 2 (the two‐way interaction model) builds on Model 1 by additionally including interactions between each aspect of the model minority stereotype predictor, GPA, and SES (parental educational attainment or household income). That is, Model 2(a) includes achievement aspect × GPA + achievement aspect × SES (parental educational attainment or household income) + GPA × SES (parental educational attainment or household income), and Model 2(b) includes mobility aspect × GPA + mobility aspect × SES (parental educational attainment or household income) + GPA × SES (parental educational attainment or household income). Similarly, Model 3 (the three‐way interaction model) builds on Model 2 by further adding the three‐way interaction of model minority stereotype, GPA, and SES (parental educational attainment or household income). That is, Model 3(a) includes achievement aspect × GPA × SES (parental educational attainment or household income), and Model 3(b) includes mobility aspect × GPA × SES (parental educational attainment or household income). These analyses were conducted separately within the Filipino American and Korean American groups. Statistically significant interaction terms (*p* < .05) were subsequently illustrated through graphical plots. Missing data were handled using maximum likelihood estimation.

### Final model selection

Analyses were performed to assess models with and without the decomposition of between‐ and within‐subjects effects of each aspect of the model minority stereotype and GPA across three different modeling approaches: (1) the direct effect model, (2) the two‐way interaction model, and (3) the three‐way interaction model. LR tests showed no significant differences between models with and without the decomposition across all three approaches. Therefore, we opted to use the models without decomposition, suggesting that any significant associations reported in this study reflect both cross‐sectional (between‐subjects) and longitudinal (within‐subjects) effects.

## RESULTS

### Descriptive statistics and the unadjusted trajectory of study variables

Table 1 presents the descriptive statistics of the study variables—mean and standard deviation for continuous variables and count and percentage for categorical variables—segmented by ethnicity over three waves. Significant differences by ethnic groups were evaluated using independent‐samples *t*‐tests. Furthermore, unadjusted trajectories of the study variables over time were analyzed using mixed‐effects regression models, treating each study variable as a separate outcome. The descriptive analysis revealed several demographic differences between groups. The Korean American group was generally younger and included fewer female participants, compared with the Filipino American group. Filipino American youth consistently had higher scores on the mobility aspect of the model minority stereotype across all three waves and were more likely to come from a family with higher parental educational attainment and income, compared with Korean American youth. Longitudinal trajectory analysis showed similar patterns of change over time for both ethnic groups in several key measures: self‐reported general health and both aspects of the model minority stereotype (achievement and mobility) significantly declined over time. While there were no significant differences in mental health outcomes between ethnic groups, both groups showed a significant increase in depressive symptoms from Wave 2 to Wave 4. Notably, a significant increase in suicidal thoughts was observed only among Filipino American youth.

### Direct effect models

The direct effect models (Model 1) for depressive symptoms and suicidal thoughts are presented in Table 2 for the models with parental educational attainment and Table 3 for the models with household income. Both tables show identical significant results. Specifically, the results regarding demographic controls revealed that female participants across both ethnic groups were more likely to experience depressive symptoms and suicidal thoughts. United States‐born Korean American youth reported higher levels of depressive symptoms than their foreign‐born counterparts. Additionally, better general health was associated with lower rates of depressive symptoms and suicidal thoughts, regardless of ethnicity. Among the achievement indicators, only GPA exhibited significant direct effects on mental health outcomes, whereas parental educational attainment did not. Specifically, a higher GPA was associated with reduced depressive symptoms and a lower likelihood of suicidal thoughts across both ethnic groups. With respect to the model minority stereotype, among Korean American youth, the achievement aspect was linked to increased depressive symptoms, and the mobility aspect was associated with decreased depressive symptoms. Among Filipino American youth, no significant associations emerged between the achievement aspect of the model minority stereotype and mental health outcomes. However, the mobility aspect of the model minority stereotype was associated with decreased depressive symptoms.

**TABLE 2:** Model 1: mixed‐effects regression results for depressive symptoms and suicidal thoughts among Filipino American and Korean American youth related to parental education.

Model 1	Filipino American youth		Korean American youth	
	Depressive symptoms	Suicidal thoughts	Depressive symptoms	Suicidal thoughts
	*b* (SE)	OR (SE)	*b* (SE)	OR (SE)
Wave	0.77**	0.22	0.31	0.66
	(0.27)	(0.32)	(0.22)	(0.83)
Baseline age	0.02	0.79^†^	0.02	0.97
	(0.03)	(0.11)	(0.02)	(0.11)
Wave × baseline age	−0.04*	1.11	−0.01	1.03
	(0.02)	(0.11)	(0.01)	(0.09)
Female (ref: male)	0.35***	2.29*	0.33***	2.52**
	(0.07)	(0.81)	(0.07)	(0.79)
Nativity (ref: foreign‐born)	−0.13	0.74	0.15*	1.23
	(0.08)	(0.27)	(0.07)	(0.39)
General health	−0.30***	0.47***	−0.23***	0.61**
	(0.04)	(0.09)	(0.03)	(0.10)
Achievement aspect	0.04	0.95	0.10**	0.91
	(0.04)	(0.19)	(0.04)	(0.18)
Mobility aspect	−0.11*	0.80	−0.10*	0.99
	(0.04)	(0.17)	(0.04)	(0.19)
GPA	−0.21***	0.36***	−0.17**	0.49*
	(0.06)	(0.10)	(0.06)	(0.14)
College degree (ref: below college)	0.08	1.10	−0.00	1.27
	(0.09)	(0.46)	(0.07)	(0.41)
*ICC*	0.34	0.40	0.45	0.43

*Note*: *ICC* indicates the proportion of (unexplained) variance of outcome measures at the subject level (level 2 in longitudinal data format). Abbreviations: *ICC*, intraclass correlation coefficient; OR, odds ratio. ***p < .001; **p < .01; *p < .05; ^†^ *p* < .1.

**TABLE 3:** Model 1: mixed‐effects regression results for depressive symptoms and suicidal thoughts among Filipino American and Korean American youth related to household income.

Model 1	Filipino American youth		Korean American youth	
	Depressive symptoms	Suicidal thoughts	Depressive symptoms	Suicidal thoughts
	*b* (SE)	OR (SE)	*b* (SE)	OR (SE)
Wave	0.74**	0.24	0.21	0.67
	(0.27)	(0.35)	(0.22)	(0.87)
Baseline age	0.02	0.77^†^	0.02	0.97
	(0.03)	(0.11)	(0.02)	(0.12)
Wave × baseline age	−0.04*	1.10	−0.01	1.03
	(0.02)	(0.11)	(0.01)	(0.09)
Female (ref: male)	0.33***	2.13*	0.34***	2.69**
	(0.07)	(0.75)	(0.07)	(0.89)
Nativity (ref: foreign‐born)	−0.11	0.82	0.17*	1.27
	(0.07)	(0.31)	(0.07)	(0.42)
General health	−0.30***	0.49***	−0.24***	0.62**
	(0.04)	(0.09)	(0.03)	(0.10)
Achievement aspect	0.03	0.95	0.10**	0.89
	(0.04)	(0.19)	(0.04)	(0.18)
Mobility aspect	−0.10*	0.79	−0.10*	1.04
	(0.04)	(0.17)	(0.04)	(0.21)
GPA	−0.21***	0.36***	−0.17**	0.50*
	(0.06)	(0.10)	(0.06)	(0.15)
Household income (ref: below $75,000)	−0.02	0.73	0.05	1.05
	(0.08)	(0.26)	(0.07)	(0.34)
*ICC*	0.34	0.41	0.45	0.46

*Note*: *ICC* indicates the proportion of (unexplained) variance of outcome measures at the subject level (level 2 in longitudinal data format). Abbreviations: *ICC*, intraclass correlation coefficient; OR, odds ratio. ***p < .001; **p < .01; *p < .05; ^†^ *p* < .1.

### Two‐way interaction models

**FIGURE 1:**
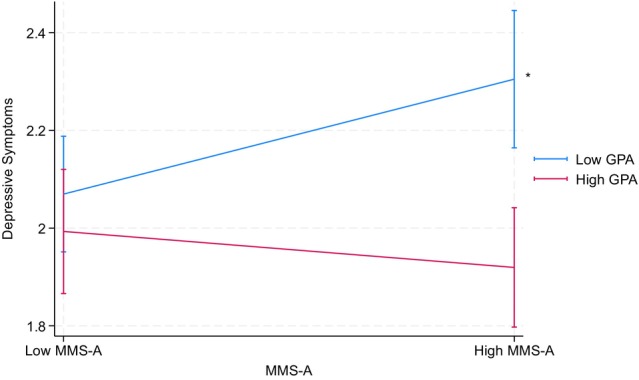
Two‐way interaction effect of the achievement aspect and GPA on depressive symptoms among Filipino American youth related to parental education. MMS‐A refers to the achievement aspect of the model minority stereotype. * Statistically significant at the .05 level.

Table 4 presents the results from Model 2(a), a mixed‐effects regression analyzing three two‐way interactions involving the achievement aspect of the model minority stereotype: achievement aspect × GPA, achievement aspect × parental educational attainment, and GPA × parental educational attainment. No significant interaction effects emerged among Korean American youth, indicating that the direct effects observed in Model 1 did not vary by GPA or parental educational attainment. However, among Filipino American youth, GPA significantly moderated the relationship between the achievement aspect and depressive symptoms (*b* = −0.20, *p* < .05). Specifically, the achievement aspect was linked to higher levels of depressive symptoms only for Filipino American youth with lower GPA, but not for those with higher GPA (see Figure 1). In contrast, no significant interactions were found between the achievement aspect and parental educational attainment on any mental health outcomes for Filipino American youth.

**TABLE 4:** Model 2(a): mixed‐effects regression results for Filipino American and Korean American youth involving the achievement aspect of the model minority stereotype related to parental education.

Model 2(a)	Filipino American youth		Korean American youth	
	Depressive symptoms	Suicidal thoughts	Depressive symptoms	Suicidal thoughts
	*b* (SE)	OR (SE)	*b* (SE)	OR (SE)
Wave	0.80**	0.25	0.31	0.68
	(0.27)	(0.37)	(0.22)	(0.86)
Baseline age	0.02	0.80^†^	0.02	0.96
	(0.03)	(0.11)	(0.02)	(0.11)
Wave × baseline age	−0.05**	1.10	−0.01	1.03
	(0.02)	(0.11)	(0.01)	(0.09)
Female (ref: male)	0.35***	2.24*	0.33***	2.53**
	(0.07)	(0.79)	(0.07)	(0.79)
Nativity (ref: foreign‐born)	−0.15^†^	0.73	0.15*	1.23
	(0.08)	(0.27)	(0.07)	(0.39)
General health	−0.29***	0.48***	−0.23***	0.60**
	(0.04)	(0.09)	(0.03)	(0.10)
Achievement aspect	0.66*	2.50	0.33	1.90
	(0.28)	(3.20)	(0.27)	(2.63)
Mobility aspect	−0.11*	0.78	−0.10**	0.98
	(0.04)	(0.17)	(0.04)	(0.19)
GPA	0.44	0.55	−0.06	0.74
	(0.28)	(0.71)	(0.27)	(1.00)
College degree (ref: below college)	−0.18	0.13	−0.28	26.88
	(0.54)	(0.31)	(0.43)	(60.42)
Achievement aspect × GPA	−0.20*	0.75	−0.05	0.84
	(0.08)	(0.27)	(0.08)	(0.33)
Achievement aspect × college degree	0.09	1.00	−0.10	0.79
	(0.09)	(0.45)	(0.07)	(0.29)
GPA × college degree	−0.01	1.89	0.09	0.51
	(0.14)	(1.22)	(0.12)	(0.28)
*ICC*	0.35	0.39	0.45	0.43

*Note*: *ICC* indicates the proportion of (unexplained) variance of outcome measures at the subject level (level 2 in longitudinal data format). Abbreviation: ICC, intraclass correlation coefficient. ***p < .001; **p < .01; *p < .05; ^†^ *p* < .1.

**FIGURE 2:**
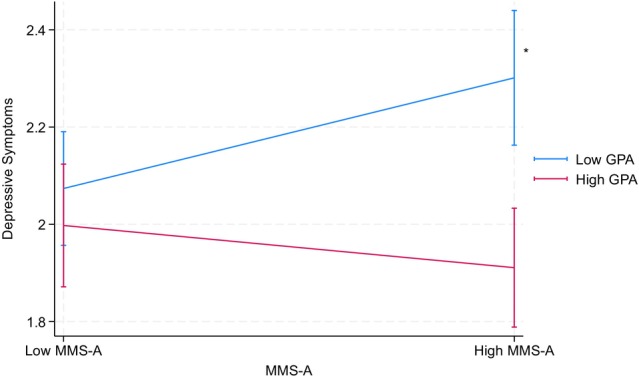
Two‐way interaction effect of the achievement aspect and GPA on depressive symptoms among Filipino American youth related to household income. MMS‐A refers to the achievement aspect of the model minority stereotype. * Statistically significant at the .05 level.

**FIGURE 3:**
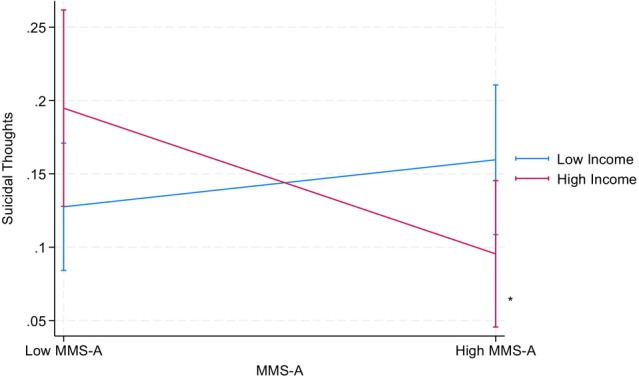
Two‐Way interaction effect of the achievement aspect and household income on suicidal thoughts among Korean American youth. MMS‐A refers to the mobility aspect of the model minority stereotype.* Statistically significant at the .05 level.

Table 5 presents the results from a parallel set of analyses replacing parental educational attainment with household income, examining the following interactions: achievement aspect × GPA, achievement aspect × household income, and GPA × household income. Among Filipino American youth, GPA significantly moderated the relationship between the achievement aspect and depressive symptoms (*b* = −0.20, *p* < .01). Specifically, the achievement aspect was linked to higher levels of depressive symptoms only for Filipino American youth with lower GPA, but not for those with higher GPA (see Figure 2). In contrast, no significant interactions were found between the achievement aspect and household income on any mental health outcomes for Filipino American youth. However, for Korean American youth, household income significantly moderated the relationship between the achievement aspect and suicidal thoughts (*b* = 0.37, *p* < .05). Specifically, the achievement aspect was linked to lower levels of suicidal thoughts only for Korean American youth with higher household income, but not for those with lower household income (see Figure 3). No significant interactions were found between the achievement aspect and GPA on any mental health outcomes for Korean American youth.

**TABLE 5:** Model 2(a): mixed‐effects regression results for Filipino American and Korean American youth involving the achievement aspect of the model minority stereotype related to household income.

Model 2(a)	Filipino American youth		Korean American youth	
	Depressive symptoms	Suicidal thoughts	Depressive symptoms	Suicidal thoughts
	*b* (SE)	OR (SE)	*b* (SE)	OR (SE)
Wave	0.78**	0.27	0.23	0.70
	(0.27)	(0.40)	(0.22)	(0.90)
Baseline age	0.02	0.79^†^	0.02	0.97
	(0.03)	(0.11)	(0.02)	(0.12)
Wave × baseline age	−0.05**	1.09	−0.01	1.02
	(0.02)	(0.11)	(0.01)	(0.09)
Female (ref: male)	0.31***	2.07*	0.33***	2.66**
	(0.07)	(0.73)	(0.07)	(0.88)
Nativity (ref: foreign‐born)	−0.12	0.79	0.17*	1.25
	(0.08)	(0.30)	(0.07)	(0.42)
General health	−0.30***	0.48***	−0.23***	0.62**
	(0.04)	(0.09)	(0.03)	(0.11)
Achievement aspect	0.78**	2.61	0.31	3.16
	(0.28)	(3.28)	(0.27)	(4.63)
Mobility aspect	−0.11**	0.80	−0.09*	1.07
	(0.04)	(0.17)	(0.04)	(0.22)
GPA	0.32	1.46	0.09	1.65
	(0.27)	(1.84)	(0.27)	(2.43)
Household income (ref: below $75,000)	−0.41	2.69	1.36**	689.41*
	(0.48)	(5.78)	(0.50)	(1752.82)
Achievement aspect × GPA	−0.20**	0.72	−0.05	0.77
	(0.08)	(0.26)	(0.08)	(0.32)
Achievement aspect × household income	−0.07	1.13	−0.10	0.37*
	(0.08)	(0.44)	(0.07)	(0.15)
GPA × household income	0.17	0.60	−0.28*	0.39
	(0.12)	(0.33)	(0.12)	(0.24)
*ICC*	0.34	0.41	0.46	0.45

*Note*: *ICC* indicates the proportion of (unexplained) variance of outcome measures at the subject level (level 2 in longitudinal data format). The odds ratio (OR) for household income was extremely large (*OR* = 689.41). To address this, we removed the two nonsignificant interaction terms and re‐estimated the model. After doing so, the OR for income decreased substantially to 26. Notably, the follow‐up interaction plot showed the same pattern observed in Figure 5. Abbreviation: ICC, intraclass correlation coefficient. ***p < .001; **p < .01; *p < .05; ^†^ *p* < .1.

**FIGURE 4:**
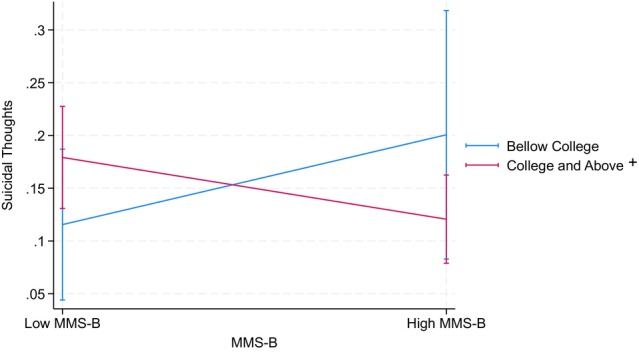
Two‐way interaction effect of the mobility aspect and parental education on suicidal thoughts among Filipino American youth. MMS‐B refers to the mobility aspect of the model minority stereotype.^+^ Statistically significant at the .1 level.

Table 6 details the findings from three two‐way interactions related to the mobility aspect of the model minority stereotype: mobility aspect × GPA, mobility aspect × parental educational attainment, and GPA × parental educational attainment. None of these interactions showed statistically significant effects on depressive symptoms or suicidal thoughts, except that mobility aspect × parental educational attainment significantly predicted suicidal thoughts among the Filipino American youth (*OR* = 0.34, *p* < .05). That is, higher mobility scores were associated with reduced suicidal thoughts when parental educational attainment was at the college level or above, whereas lower mobility scores were linked to increased suicidal thoughts. Although neither simple slope reached statistical significance at the .05 level (one was marginally significant at the .10 level), their directions differed significantly from each other (see Figure 4).

**TABLE 6:** Model 2(b): mixed‐effects regression results for Filipino American and Korean American youth involving the mobility aspect of the model minority stereotype related to parental education.

Model 2(b)	Filipino American youth		Korean American youth	
	Depressive symptoms	Suicidal Thoughts	Depressive symptoms	Suicidal Thoughts
	*b* (SE)	OR (SE)	*b* (SE)	OR (SE)
Wave	0.78**	0.19	0.30	0.69
	(0.27)	(0.28)	(0.22)	(0.88)
Baseline age	0.02	0.78^†^	0.02	0.96
	(0.03)	(0.11)	(0.02)	(0.11)
Wave × baseline age	−0.05**	1.12	−0.01	1.03
	(0.02)	(0.11)	(0.01)	(0.09)
Female (ref: male)	0.35***	2.27*	0.33***	2.53**
	(0.07)	(0.80)	(0.07)	(0.08)
Nativity (ref: foreign‐born)	−0.14^†^	0.74	0.15*	1.25
	(0.08)	(0.24)	(0.07)	(0.40)
General health	−0.30***	0.47***	−0.23***	0.60**
	(0.04)	(0.09)	(0.03)	(0.10)
Achievement aspect	0.03	0.93	0.10**	0.91
	(0.04)	(0.19)	(0.04)	(0.18)
Mobility aspect	0.55^†^	1.09	−0.14	0.63
	(0.31)	(1.64)	(0.27)	(0.79)
GPA	0.21	0.14	−0.25	0.50
	(0.27)	(0.19)	(0.22)	(0.54)
College degree (ref: below college)	0.59	2.06	−0.30	8.93
	(0.56)	(5.32)	(0.45)	(18.81)
GPA × mobility aspect	−0.15^†^	1.18	0.01	1.12
	(0.09)	(0.49)	(0.08)	(0.41)
GPA × college degree	−0.00	1.94	0.08	0.52
	(0.14)	(1.25)	(0.12)	(0.29)
Mobility aspect × college degree	−0.18^†^	0.34*	0.01	1.12
	(0.10)	(0.18)	(0.07)	(0.41)
*ICC*	0.33	0.39	0.45	0.43

*Note*: *ICC* indicates the proportion of (unexplained) variance of outcome measures at the subject level (level 2 in longitudinal data format). Abbreviation: ICC, intraclass correlation coefficient. ***p < .001; **p < .01; *p < .05; ^†^ *p* < .1.

**FIGURE 5:**
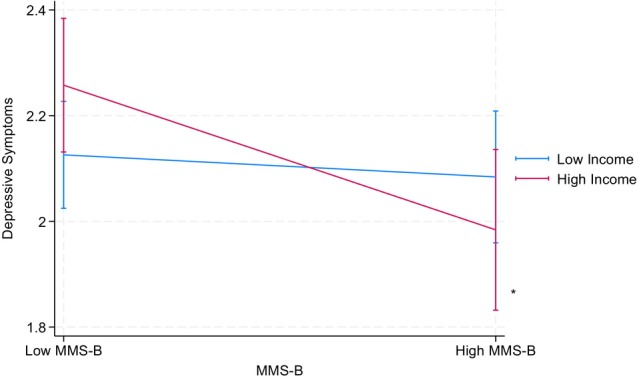
Two‐way interaction effect of the mobility aspect and household income on depressive symptoms among Korean American youth. MMS‐B refers to the mobility aspect of the model minority stereotype.* Statistically significant at the .05 level.

Table 7 summarizes the results from parallel analyses using household income in place of parental educational attainment, examining the following interactions: mobility aspect × GPA, mobility aspect × household income, and GPA × household income. None of these interactions showed statistically significant effects on depressive symptoms or suicidal thoughts, except that mobility aspect × household income significantly predicted depressive symptoms among the Korean American youth (*b* = −0.16, *p* < .05). That is, higher mobility scores were associated with reduced depressive symptoms among families with higher household income, whereas we found no statistically significant association between mobility scores and reduced depressive symptoms among lower‐income families (see Figure 5).

**TABLE 7:** Model 2(b): mixed‐effects regression results for Filipino American and Korean American Youth involving the mobility aspect of the model minority stereotype related to household income.

Model 2 (b)	Filipino American youth		Korean American youth	
	Depressive symptoms	Suicidal thoughts	Depressive symptoms	Suicidal thoughts
	*b* (SE)	OR (SE)	*b* (SE)	OR (SE)
Wave	0.76**	0.22	0.24	0.65
	(0.27)	(0.34)	(0.22)	(0.85)
Baseline age	0.02	0.77^†^	0.02	0.97
	(0.03)	(0.11)	(0.02)	(0.12)
Wave × baseline age	−0.04*	1.11	−0.01	1.03
	(0.02)	(0.11)	(0.01)	(0.09)
Female (ref: male)	0.32***	2.07*	0.33***	2.72**
	(0.07)	(0.75)	(0.07)	(0.90)
Nativity (ref: foreign‐born)	−0.10	0.83	0.17*	1.27
	(0.08)	(0.32)	(0.07)	(0.42)
General health	−0.30***	0.47***	−0.23***	0.62**
	(0.04)	(0.09)	(0.03)	(0.10)
Achievement aspect	0.03	0.88	0.10**	0.89
	(0.04)	(0.19)	(0.04)	(0.18)
Mobility aspect	0.41	1.04	−0.06	0.75
	(0.31)	(1.54)	(0.27)	(0.98)
GPA	0.04	0.39	−0.10	0.52
	(0.26)	(0.49)	(0.22)	(0.58)
Household income (ref: below $75,000)	−0.37	25.40	1.37**	14.62
	(0.48)	(56.88)	(0.47)	(33.20)
GPA × mobility aspect	−0.13	1.07	0.01	1.09
	(0.09)	(0.44)	(0.08)	(0.41)
GPA × household income	0.17	0.66	−0.26*	0.42
	(0.12)	(0.37)	(0.13)	(0.26)
Mobility aspect × household income	−0.09	0.45^†^	−0.16*	1.13
	(0.09)	(0.20)	(0.07)	(0.42)
*ICC*	0.35	0.43	0.46	0.45

*Note*: *ICC* indicates the proportion of (unexplained) variance of outcome measures at the subject level (level 2 in longitudinal data format). Abbreviation: ICC, intraclass correlation coefficient. ***p < .001; **p < .01; *p < .05; ^†^ *p* < .1.

### Three‐way interaction models

**FIGURE 6:**
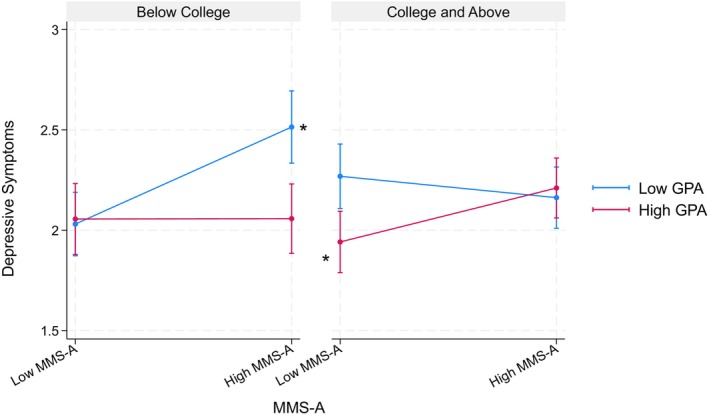
Three‐way interaction effect of the achievement aspect, gpa, and parental education on depressive symptoms among Korean American youth. MMS‐A refers to the achievement aspect of the model minority stereotype.* Statistically significant at the .05 level.

Table 8 details findings from three‐way interaction models examining the achievement aspect of the model minority stereotype in relation to parental educational attainment. Among Filipino American youth, none of these interactions reached statistical significance. However, among the Korean American youth, we found a significant interaction of the achievement aspect × GPA × parental educational attainment (*b* = 0.62, *p* < .001) on depressive symptoms. Specifically, when parents held a college degree or higher, the achievement aspect was significantly associated with more depressive symptoms only among those with a higher GPA. Conversely, among youth whose parents had a high school education or less, the achievement aspect was related to greater depressive symptoms only among those with lower GPA (see Figure 6).

**TABLE 8:** Model 3(a): mixed‐effects regression results for Filipino American and Korean American youth involving the achievement aspect of the model minority stereotype related to parental education.

Model 3(a)	Filipino American youth		Korean American youth	
	Depressive symptoms	Suicidal thoughts	Depressive Symptoms	Suicidal thoughts
	*b* (SE)	OR (SE)	*b* (SE)	OR (SE)
Wave	0.81**	0.25	0.33	0.72
	(0.27)	(0.37)	(0.22)	(0.91)
Baseline age	0.03	0.79^†^	0.03	0.97
	(0.03)	(0.11)	(0.02)	(0.12)
Wave × baseline age	−0.05**	1.10	−0.02	1.02
	(0.02)	(0.11)	(0.01)	(0.09)
Female (ref: male)	0.34***	2.27*	0.33***	2.56**
	(0.07)	(0.81)	(0.07)	(0.81)
Nativity (ref: foreign‐born)	−0.15^†^	0.73	0.15*	1.23
	(0.08)	(0.27)	(0.07)	(0.39)
General health	−0.30***	0.48***	−0.23***	0.60**
	(0.04)	(0.09)	(0.03)	(0.10)
Achievement aspect	−0.03	5.46	1.39***	15.72
	(0.57)	(14.48)	(0.37)	(33.49)
Mobility aspect	−0.11*	0.79	−0.11**	0.98
	(0.04)	(0.17)	(0.04)	(0.19)
GPA	−0.21	1.14	0.94**	9.66
	(0.55)	(2.84)	(0.36)	(20.08)
College degree (ref: below college)	−3.05	2.96	7.32***	2.75e+07^†^
	(2.14)	(28.48)	(1.82)	(2.74e+07)
Achievement aspect × GPA	0.00	0.60	−0.35***	0.45
	(0.16)	(0.46)	(0.11)	(0.28)
Achievement aspect × college degree	0.98	0.37	−2.29***	0.01
	(0.65)	(1.11)	(0.53)	(0.04)
GPA × college degree	0.83	0.27	−2.00***	0.01
	(0.62)	(2.10)	(0.52)	(0.03)
Achievement aspect × GPA × college degree	−0.26	1.34	0.62***	3.28
	(0.19)	(1.17)	(0.15)	(2.71)
*ICC*	0.35	0.39	0.45	0.43

*Note*: *ICC* indicates the proportion of (unexplained) variance of outcome measures at the subject level (level 2 in longitudinal data format). Abbreviation: ICC, intraclass correlation coefficient. ***p < .001; **p < .01; *p < .05; ^†^ *p* < .1.

**FIGURE 7:**
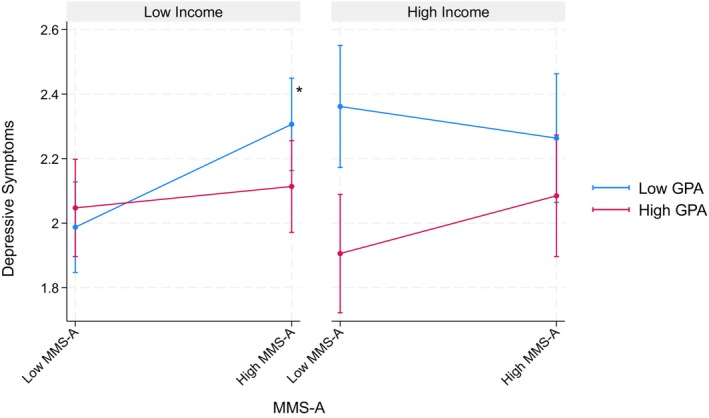
Three‐way interaction effect of the achievement aspect, gpa, and household income on depressive symptoms among Korean American youth. MMS‐A refers to the achievement aspect of the model minority stereotype.* Statistically significant at the .05 level.

Table 9 shows findings from three‐way interaction models investigating the achievement aspect of the model minority stereotype in relation to household income. Among Filipino American youth, none of these interactions reached statistical significance. However, among the Korean American youth, we found a significant interaction of the achievement aspect × GPA × household income (*b* = 0.39, *p* < .001) on depressive symptoms. Specifically, among youth from lower‐income households, the achievement aspect was significantly associated with more depressive symptoms only among those with lower GPAs. However, no significant associations were found among youth from higher‐income families (see Figure 7).

**TABLE 9:** Model 3(a): mixed‐effects regression results for Filipino American and Korean American youth involving the achievement aspect of the model minority stereotype related to household income.

Model 3(a)	Filipino American youth		Korean American youth	
	Depressive symptoms	Suicidal thoughts	Depressive symptoms	Suicidal thoughts
	*b* (SE)	OR (SE)	*b* (SE)	OR (SE)
Wave	0.81**	0.27	0.22	0.71
	(0.27)	(0.40)	(0.22)	(0.92)
Baseline age	0.02	0.78^†^	0.02	0.97
	(0.03)	(0.11)	(0.02)	(0.12)
Wave × baseline age	−0.05**	1.09	−0.01	1.02
	(0.02)	(0.11)	(0.01)	(0.09)
Female (ref: male)	0.32***	2.07*	0.33***	2.68**
	(0.07)	(0.73)	(0.07)	(0.89)
Nativity (ref: foreign‐born)	−0.12	0.79	0.17*	1.25
	(0.08)	(0.30)	(0.07)	(0.42)
General health	−0.30***	0.48***	−0.23***	0.62**
	(0.04)	(0.09)	(0.03)	(0.10)
Achievement aspect	0.12	4.04	0.77*	5.92
	(0.50)	(8.79)	(0.33)	(10.88)
Mobility aspect	−0.12**	0.81	−0.10*	1.06
	(0.04)	(0.18)	(0.04)	(0.21)
GPA	−0.32	2.24	0.54^†^	3.06
	(0.48)	(4.78)	(0.33)	(5.61)
Household income (ref: below $75,000)	−3.45^†^	21.47	5.81**	2.86e+05^†^
	(1.97)	(186.46)	(1.91)	(2.96e+06)
Achievement aspect × GPA	−0.01	0.63	−0.18^†^	0.64
	(0.14)	(0.40)	(0.09)	(0.33)
Achievement aspect × household income	0.87	0.60	−1.45**	0.06
	(0.59)	(1.56)	(0.56)	(0.18)
GPA × household income	1.06^†^	0.32	−1.56**	0.07
	(0.57)	(0.82)	(0.54)	(0.20)
Achievement aspect × GPA × household income	−0.27	1.21	0.39***	1.72
	(0.17)	(0.93)	(0.16)	(1.56)
*ICC*	0.34	0.40	0.46	0.45

*Note*: *ICC* indicates the proportion of (unexplained) variance of outcome measures at the subject level (level 2 in longitudinal data format). Abbreviation: ICC, intraclass correlation coefficient. ***p < .001; **p < .01; *p < .05; ^†^ *p* < .1.

**FIGURE 8:**
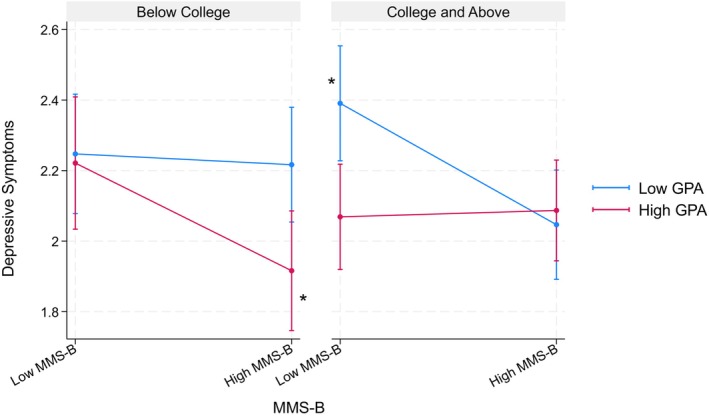
Three‐way interaction effect of the mobility aspect, gpa, and parental education on depressive symptoms among Korean American youth. MMS‐B refers to the mobility aspect of the model minority stereotype.* Statistically significant at the .05 level.

Table 10 details findings from three‐way interaction models examining the mobility aspect of the model minority stereotype with respect to parental educational attainment. Similar to three‐way interactions for the achievement aspect models, none of these interactions reached statistical significance for the Filipino American youth. However, among the Korean American youth, we found significant interaction of the mobility aspect × GPA × parental educational attainment (*b* = 0.48, *p* < .01) on depressive symptoms. Specifically, when parents held a college degree or higher, the mobility aspect was significantly associated with fewer depressive symptoms only among those with a lower GPA. On the other hand, among youth whose parents had a high school education or less, the mobility aspect was significantly related to fewer depressive symptoms only among those with higher GPA (see Figure 8).

**TABLE 10:** Model 3(b): mixed‐effects regression results for Filipino American and Korean American youth involving the mobility aspect of the model minority stereotype related to parental education.

Model 3(b)	Filipino American youth		Korean American youth	
	Depressive symptoms	Suicidal thoughts	Depressive symptoms	Suicidal thoughts
	*b* (SE)	OR (SE)	*b* (SE)	OR (SE)
Wave	0.80**	0.18	0.31	0.72
	(0.27)	(0.28)	(0.22)	(0.92)
Baseline age	0.02	0.78^†^	0.03	0.98
	(0.03)	(0.11)	(0.02)	(0.12)
Wave × baseline age	−0.05**	1.12	−0.01	1.02
	(0.02)	(0.11)	(0.01)	(0.09)
Female (ref: male)	0.35***	2.28*	0.33***	2.50**
	(0.07)	(0.81)	(0.07)	(0.80)
Nativity (ref: foreign‐born)	−0.14^†^	0.74	0.16*	1.27
	(0.08)	(0.27)	(0.07)	(0.41)
General health	−0.30***	0.47***	−0.23***	0.60**
	(0.04)	(0.09)	(0.03)	(0.10)
Achievement aspect	0.03	0.93	0.11**	0.92
	(0.04)	(0.19)	(0.04)	(0.18)
Mobility aspect	0.26	2.20	0.06^†^	3.31
	(0.64)	(6.82)	(0.35)	(5.70)
GPA	−0.22	0.24	0.35	2.00
	(0.53)	(0.68)	(0.29)	(2.93)
College degree (ref: below college)	−0.46	18.31	4.20**	4.15e+05^†^
	(2.11)	(197.83)	(1.49)	(3.07e+06)
Mobility aspect × GPA	−0.06	0.99	−0.20*	0.69
	(0.18)	(0.95)	(0.10)	(0.34)
Mobility aspect × college degree	0.19	0.16	−1.65**	0.02
	(0.73)	(0.59)	(0.53)	(0.05)
GPA × college degree	0.30	1.02	−1.21**	0.02^†^
	(0.60)	(3.20)	(0.42)	(0.05)
Mobility aspect × GPA × college degree	−0.11	1.25	0.48**	3.20
	(0.21)	(1.35)	(0.15)	(2.43)
*ICC*	0.33	0.40	0.45	0.44

*Note*: *ICC* indicates the proportion of (unexplained) variance of outcome measures at the subject level (level 2 in longitudinal data format). Abbreviation: ICC, intraclass correlation coefficient. ***p < .001; **p < .01; *p < .05; ^†^ *p* < .1.

**FIGURE 9:**
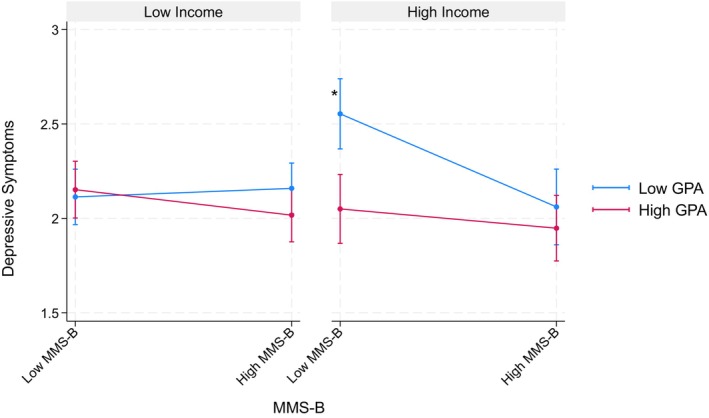
Three‐way interaction effect of the mobility aspect, gpa, and household income on depressive symptoms among Korean American youth. MMS‐B refers to the mobility aspect of the model minority stereotype.* Statistically significant at the .05 level.

Table 11 details findings from three‐way interaction models investigating the mobility aspect of the model minority stereotype regarding household income. Similar to three‐way interactions for the mobility aspect models related to parental educational attainment, none of these interactions reached statistical significance for the Filipino American youth. However, among the Korean American youth, we found a significant interaction of the mobility aspect × GPA × household income (*b* = 0.43, *p* < .01) on depressive symptoms. Specifically, among youth from a higher‐income family, the mobility aspect was significantly associated with fewer depressive symptoms only among those with lower GPA. However, no significant associations were found among youth from lower‐income families (see Figure 9).

**TABLE 11:** Model 3(b): mixed‐effects regression results for Filipino American and Korean American Youth involving the mobility aspect of the model minority stereotype related to household income.

Model 3(b)	Filipino American youth		Korean American youth	
	Depressive symptoms	Suicidal thoughts	Depressive symptoms	Suicidal thoughts
	*b* (SE)	OR (SE)	*b* (SE)	OR (SE)
Wave	0.77**	0.22	0.21	0.64
	(0.27)	(0.34)	(0.22)	(0.84)
Baseline age	0.02	0.77^†^	0.02	0.97
	(0.03)	(0.11)	(0.02)	(0.12)
Wave × baseline age	−0.05**	1.11	−0.01	1.03
	(0.02)	(0.11)	(0.01)	(0.09)
Female (ref: male)	0.33***	2.07*	0.33***	2.73**
	(0.07)	(0.76)	(0.07)	(0.90)
Nativity (ref: foreign‐born)	−0.11	0.82	0.17*	1.27
	(0.08)	(0.32)	(0.07)	(0.42)
General health	−0.30***	0.47***	−0.23***	0.62**
	(0.04)	(0.10)	(0.03)	(0.10)
Achievement aspect	0.03	0.88	0.10*	0.88
	(0.04)	(0.19)	(0.04)	(0.18)
Mobility aspect	0.04	0.95	0.44	0.94
	(0.64)	(2.86)	(0.32)	(1.46)
GPA	−0.26	0.36	0.29	0.63
	(0.53)	(0.90)	(0.26)	(0.81)
Household income (ref: below $75,000)	−1.69	18.37	5.31***	112.07
	(2.08)	(180.30)	(1.53)	(860.69)
Mobility aspect × GPA	−0.02	1.10	−0.13*	1.02
	(0.18)	(0.94)	(0.09)	(0.46)
Mobility aspect × household income	0.38	0.51	−1.66**	0.52
	(0.73)	(1.73)	(0.56)	(1.46)
GPA × household income	0.55	0.72	−1.38***	0.23
	(0.59)	(2.05)	(0.43)	(0.52)
Mobility aspect × GPA × household income	−0.14	0.97	0.43**	1.25
	(0.21)	(0.95)	(0.16)	(1.03)
*ICC*	0.34	0.43	0.46	0.46

*Note*: *ICC* indicates the proportion of (unexplained) variance of outcome measures at the subject level (level 2 in longitudinal data format). Abbreviation: ICC, intraclass correlation coefficient. ***p < .001; **p < .01; *p < .05; ^†^ *p* < .1.

### Alternative models

As a sensitivity check, we examined whether the moderating effect of GPA further varies by the youth's self‐reported subjective family SES (each aspect of the stereotype × GPA × subjective family SES). Subjective family SES was assessed using a single five‐point Likert‐scale item (lower class, lower middle class, middle class, upper middle class, upper class) developed from the Midwest Longitudinal Study of Asian American Families and was subsequently dichotomized into a binary variable, with 1 indicating middle class or higher (middle class, upper middle class, and upper class) and 0 indicating below middle class (lower class and lower middle class). The results showed no statistically significant three‐way interaction effects across all models, irrespective of the ethnic groups involved, except for suicidal thoughts among Korean American youth (the achievement aspect × GPA × subjective family SES). However, a follow‐up analysis showed that none of the slopes reached statistical significance, indicating a lack of meaningful interpretation.

## DISCUSSION

Asian Americans are often labeled as a “model minority,” a stereotype characterized by high academic and socioeconomic success that is commonly attributed to a cultural prioritization of diligence, education, and meritocratic ideals. Despite these stereotypes, Asian Americans face racism and multifaceted challenges. For example, Asian Americans show a bimodal distribution in SES and youth adjustments, but those who struggle may be overshadowed by high‐achieving individuals (Cook et al., 2011; Lee, 2015). Existing conceptual (Festinger, 1957) and empirical (Yoo et al., 2015) research suggests that a mismatch between the high expectations imposed by the model minority stereotype and Asian Americans' lived experiences—particularly when these experiences fall short of these expectations—can lead to significant mental discomfort. Despite these insights, little research has examined how internalizing the model minority stereotype is associated with the mental health of Asian American youth and how this relationship differs based on individual academic achievement and family SES, particularly using longitudinal data. To address these gaps, this study explored how the relations between internalization of the model minority stereotype and mental health outcomes vary by GPA, parental educational attainment, and household income among two major Asian American subgroups: Filipino Americans and Korean Americans. The findings underscore the varying impact of the model minority stereotype across ethnic groups, highlighting the importance of considering sociocultural contexts when understanding Asian American mental health. Moreover, the analysis indicates that decomposition of between‐ and within‐subjects effects for time‐varying variables was unnecessary, suggesting that the significant associations found in the current study would hold at both the between‐subjects level (differences across individuals) and the within‐subjects level (changes within individuals over time)—thereby strengthening support for the robustness of the study relationships.

### Effects of GPA, parental educational attainment, and household income on mental health

Regarding demographic control variables, female participants (Adams‐Prassl et al., 2022; Afifi, 2007; Kuehner, 2003), U.S.‐born participants (Alvarez et al., 2019; Budhwani et al., 2015), and those reporting poorer general health (Alonzi et al., 2020; Prince et al., 2007) tended to experience worse mental health outcomes compared to their male, foreign‐born, and healthier peers, consistent with prior research. As anticipated, even after accounting for these known demographic controls, GPA remained a significant determinant of mental health outcomes among young Asian Americans, consistent with existing literature (Whaley & Noel, 2013). This finding underscores the critical need to support academically struggling students, not only to aid their educational attainment but also to enhance their overall mental well‐being. Previous research indicates that teachers are less likely to perceive Asian American youth as struggling academically, even when students have poor academic performance, and barriers to communication between families and schools further preclude students' access to support (Cherng, 2016; Wing, 2007; Yeh et al., 2008).

Conversely, the findings show minimal effects of parental educational attainment and household income on mental health outcomes, which is consistent with the existing literature (Qin et al., 2017). The nonsignificant findings for the relationship between SES and Asian American youth mental health outcomes in our study may stem from several methodological and contextual factors. First, SES may not influence youth developmental outcomes directly, but rather indirectly—for example, through family processes that could either nurture or hinder maximization of developmental potential (Benner & Kim, 2010)—or in interaction with other variables, as reflected in our interaction models. Moreover, existing literature suggests that the effects of SES may not operate in the same way for Asian American youth as for other racial groups (Liu & Xie, 2016) and may vary across ethnic subgroups, a pattern also evident in our findings and elaborated on in the next section.

### Model minority stereotype and mental health

The findings underscore the complex, multidimensional nature of the model minority stereotype and its varying impact across different Asian American subgroups. As expected (Park et al., 2021), internalizing the achievement aspect of the stereotype was found to be detrimental, leading to increased depressive symptoms among Korean American youth. In contrast, the mobility aspect of the stereotype appeared to serve as a protective factor, mitigating mental health struggles.

Importantly, these effects varied depending on the youths' academic and socioeconomic achievements. Consistent with our hypotheses for the two‐way interaction model, higher household income appeared to buffer against the detrimental effects of the achievement aspect of the model minority stereotype on suicidal thoughts. Specifically, while the achievement aspect was not significantly associated with suicidal thoughts in the direct effect model, it became protective among Korean American youth from high‐income households. This pattern aligns with prior literature suggesting that internalization of the model minority stereotype may differentially shape mental health outcomes (Park et al., 2021). While internalization of the stereotype may heighten depressive symptoms, it may simultaneously constrain progression toward more severe or action‐oriented expressions of distress, such as suicidal ideation, in efforts to maintain the appearance of fulfilling model minority expectations—consistent with evidence linking the model minority stereotype to lower levels of externalizing behavioral outcomes (Park et al., 2021). Similarly, the mobility aspect was associated with fewer depressive symptoms only among Korean American youth from high‐income households, consistent with our expectations.

The three‐way interaction models revealed additional complexities. Regarding the achievement aspect of the stereotype, and consistent with our hypothesis, youth who were least aligned with the stereotype—those with both low GPA and low family SES (low parental educational attainment or low household income)—were especially vulnerable to its harmful effects on depressive symptoms. Contrary to our expectations, even youth who appeared most aligned with the stereotype—those with high GPA and high parental educational attainment—also experienced increased depressive symptoms when they highly internalized the achievement aspect. However, as shown in Figures 6 and 7, for those with high parental educational attainment or high household income, depressive symptoms were significantly lower among those with higher GPAs than among those with lower GPAs when internalization of the achievement aspect was low. Once youth with high GPAs strongly internalized the achievement aspect, the mental health benefits of high academic performance appeared to diminish. This pattern suggests that internalizing a high achievement stereotype may generate additional pressure to sustain such performance, undermining the otherwise protective effect of academic success. For Korean American youth—who are frequently subject to model minority stereotyping experiences and may also face elevated expectations from highly educated parents (Lee et al., 2009)—internalization of the achievement aspect may amplify both internal and external pressures. These compounded demands may contribute to worsening mental health, even among those who seem to be excelling academically and socioeconomically.

Regarding the mobility aspect of the model minority stereotype, the three‐way interaction model for Korean American youth revealed patterns that diverged from our initial hypotheses. Specifically, for Korean American youth whose academic performance and family SES were aligned—whether both high or both low—the mobility aspect did not appear to impact their mental health. However, the findings were notably different for youth whose academic performance diverged from their family's educational background. That is, among youth with high GPA but low parental educational attainment, the mobility aspect was significantly associated with fewer depressive symptoms (see Figure 8). It may be the case that belief in socioeconomic advancement may reduce psychological distress (Yoo et al., 2015), particularly when youth perceive themselves as exceeding their parents' educational standing. In such cases, internalizing the mobility aspect may affirm their upward trajectory and align with lived experiences, thereby reducing dissonance and supporting well‐being.

Conversely and unexpectedly, for youth with low GPA but high parental educational attainment or high household income, the mobility aspect was also associated with fewer depressive symptoms. However, as shown in Figures 8 and 9, those with lower GPAs exhibited significantly higher depressive symptoms than their high‐achieving peers when this group reported low internalization of the mobility aspect. This group may experience distress from not meeting the high expectations set by their parents with high educational attainment or high household income (Lee, 2015; Lee et al., 2009). When these youth highly internalized the belief in meritocratic mobility, however, the difference in depressive symptoms between low‐ and high‐GPA youth was no longer statistically significant. In this context, belief in upward mobility may serve as a compensatory psychological resource, offering hope and reducing the sense of failure or inadequacy (Yoo et al., 2015). That is, even if youth are currently underperforming relative to their parental benchmarks, internalizing the mobility aspect may provide a future‐oriented framework that buffers against depressive symptoms.

Among Filipino American youth, as expected (Yoo et al., 2015), the mobility aspect of the model minority stereotype was significantly associated with fewer depressive symptoms in the direct effect model, and we confirmed in the two‐way interaction model that higher mobility scores were associated with fewer suicidal thoughts only among youth whose parents held a college degree or higher. Conversely, the achievement aspect did not exhibit significant direct effects. Yet, it emerged as a significant predictor of depressive symptoms when considered in interaction with academic performance. Specifically, supporting our hypothesis, the achievement aspect was associated with more depressive symptoms only among Filipino American youth with lower GPA, suggesting that the internalization of the achievement aspect of the stereotype may exacerbate psychological distress when academic performance does not align with the stereotype's expectations.

In summary, the current study did not find direct effects of the model minority stereotype on Filipino American youth. However, when interactions were considered, lower GPA intensified the harmful impact of the achievement aspect of the stereotype on depressive symptoms for this ethnic group. This suggests that for Filipino American youth, who may not typically be perceived as embodying the model minority stereotype to the extent that Korean American youth are, the stereotype's effects are less pronounced overall (as shown in nonsignificant roles of the model minority stereotype in the direct effect model) but are still significant for those not meeting high academic expectations. Consistent with cognitive dissonance theory (Festinger, 1957), internalizing the model minority stereotype, particularly the achievement aspect, can become a source of psychological stress for those with lower academic performance, whereas it may have fewer depressive effects on those who meet these expectations.

This finding is particularly significant given that Filipino American students often report struggling more with academic performance, compared with other Asian American subgroups (Choi, 2008). These academic challenges are further complicated by cultural and identity factors, as demonstrated in qualitative research on Filipino American graduate students' experiences with the model minority stereotype (Nadal et al., 2010). The study findings revealed that Filipino American students often feel isolated as they struggle to identify with the broader Asian American label and experience mental distress while trying to achieve academic success without adequate academic and community support. Thus, Filipino American students may experience heightened cognitive dissonance due to their academic struggles combined with insufficient academic support, potentially exacerbating their mental health challenges. This pattern is particularly concerning given that Filipino Americans consistently report poorer mental health outcomes compared with other Asian American subgroups (Lui et al., 2024).

### Limitations and implications

Several limitations of this study must be acknowledged. The sample was limited to Filipino American and Korean American youth, and the findings may not be generalizable to other Asian American subgroups, which have distinct experiences. Additionally, the study was conducted in the Midwest and may not fully represent the diverse experiences of Asian Americans across the United States. The nonrandom sampling method could further limit the study's generalizability, although the sampling campaign was extensive and multifaceted in the target areas. Also, GPA scores were self‐reported and thus subject to recall and social desirability biases. Additionally, GPA was measured differently over time: When participants were 17 years old and younger, the survey asked for letter grades in English, math, social studies, and science, which were averaged across subjects; when participants became 18 years old or entered college, the survey asked for their overall GPA. While this shift in measurement may introduce some inconsistency, it is unlikely to meaningfully bias our findings for two reasons. First, the focus on within‐person changes in GPA over time (i.e., how deviations from a participant's own average GPA predict mental health outcomes) inherently controls for individual‐level reporting tendencies and reduces the impact of between‐wave measurement differences. Second, the between‐person effects of GPA represent participants' average GPA across all waves, which smooths out inconsistencies that may result from the different measurement methods. Given that our findings indicate the associations hold at both the within‐ and between‐subjects levels, this reinforces the validity of the two points above.

Despite these limitations, this study makes several important contributions to understanding Asian American youth mental health. It is the first to examine how GPA, parental educational attainment, and household income moderate the effects of the model minority stereotype on mental health outcomes at the Asian American subgroup level using longitudinal data. By revealing distinct patterns of stereotype effects between Filipino American and Korean American youth, it highlights the importance of considering ethnic differences when studying Asian American mental health. These findings have important implications for practitioners: Mental health professionals need to develop approaches that recognize the influence of distinct ethnic backgrounds and academic contexts on racial experiences among Asian American youth. Particular attention should be paid to academically struggling students, who may be more vulnerable to the negative effects of internalized stereotypes. However, practitioners should also recognize that high academic achievement does not protect against mental health challenges, as demonstrated by the detrimental effects of the achievement aspect on Korean American youth with both high academic performance and high household SES. This suggests the need for universal yet tailored mental health screening and support, rather than assuming that high‐achieving Asian American students are immune to mental health concerns.

## CONCLUSION

This study advances our understanding of how two aspects of the internalized model minority stereotype are associated with mental health outcomes among Asian American youth by examining their complex interactions with academic performance and SES. The findings demonstrate that the model minority stereotype exerts a multifaceted impact on the mental health of Asian American youth, with distinct patterns across ethnic backgrounds and across different levels of academic performance and SES. Among Korean American youth, the achievement aspect of the stereotype was associated with increased depressive symptoms, particularly when the youth's academic performance and SES were congruent at either similarly high or low levels, whereas it was protective against suicidal thoughts for those with high household income. In contrast, the mobility aspect served as a protective factor against depressive symptoms, especially when academic performance and SES were misaligned. For Filipino American youth, the achievement aspect was significantly predictive of increased depressive symptoms only among those with low GPA, while the mobility aspect was significantly associated with fewer depressive symptoms overall and fewer suicidal thoughts when parental educational attainment was high. These results underscore the importance of considering how the psychological effects of the two aspects of the model minority stereotype vary across ethnicity, academic achievement, and socioeconomic standings. Future research should continue to investigate the interplay of stereotypes, academic achievement, and SES to identify pathways that support mental well‐being within culturally responsive frameworks. A broader understanding of stereotype impacts can inform policies and practices aimed at addressing the unique challenges Asian American youth face in educational and social settings.

## AUTHOR CONTRIBUTIONS

**Michael Park:** Conceptualization; writing – original draft; methodology; supervision. **Bryan Gu:** Writing – review and editing; writing – original draft. **Yoonsun Choi:** Funding acquisition; writing – review and editing. **Hyung Chol Yoo:** Conceptualization; writing – review and editing. **Yuanyuan Yang:** Formal analysis; writing – original draft.

## FUNDING INFORMATION

This work was supported by the grants from the Eunice Kennedy Shriver National Institute of Child Health and Human Development, R01HD073200 (PI: YC) and Russell Sage Foundation, 2005–24450 (PI: YC).

## CONFLICT OF INTEREST STATEMENT

The authors report no conflict of interests.

## ETHICS APPROVAL STATEMENT

This study was conducted in compliance with ethical standards. All procedures of the study, including data collection and analyses, were approved by the Institutional Review Board of the University of Chicago (IRB13‐0027‐CR013; Approval Date: 12/10/24; expiration date: 12/9/25) to ensure the proper protection of human subjects, including confidentiality of the data.

## INFORMED CONSENT

All study participants provided thorough informed consent and assent.

## Data Availability

The datasets analyzed in the current study are not publicly available but can be made available from YC if certain conditions are met.
